# Improved cytokine–receptor interaction prediction by exploiting the negative sample space

**DOI:** 10.1186/s12859-020-03835-5

**Published:** 2020-10-31

**Authors:** Abhigyan Nath, André Leier

**Affiliations:** 1 Department of Biochemistry, Pt. Jawahar Lal Nehru Memorial Medical College, Raipur, 492001 India; 2 Department of Genetics, Department of Cell Developmental and Integrative Biology, School of Medicine, University of Alabama at Birmingham, Birmingham, AL USA

**Keywords:** Cytokine–receptor interaction, Negative sample space, Deep autoencoders, Random forest, *K*-means

## Abstract

**Background:**

Cytokines act by binding to specific receptors in the plasma membrane of target cells. Knowledge of cytokine–receptor interaction (CRI) is very important for understanding the pathogenesis of various human diseases—notably autoimmune, inflammatory and infectious diseases—and identifying potential therapeutic targets. Recently, machine learning algorithms have been used to predict CRIs. “Gold Standard” negative datasets are still lacking and strong biases in negative datasets can significantly affect the training of learning algorithms and their evaluation. To mitigate the unrepresentativeness and bias inherent in the negative sample selection (non-interacting proteins), we propose a clustering-based approach for representative negative sample selection.

**Results:**

We used deep autoencoders to investigate the effect of different sampling approaches for non-interacting pairs on the training and the performance of machine learning classifiers. By using the anomaly detection capabilities of deep autoencoders we deduced the effects of different categories of negative samples on the training of learning algorithms. Random sampling for selecting non-interacting pairs results in either over- or under-representation of hard or easy to classify instances. When *K*-means based sampling of negative datasets is applied to mitigate the inadequacies of random sampling, random forest (RF) together with the combined feature set of atomic composition, physicochemical-2grams and two different representations of evolutionary information performs best. Average model performances based on leave-one-out cross validation (loocv) over ten different negative sample sets that each model was trained with, show that RF models significantly outperform the previous best CRI predictor in terms of accuracy (+ 5.1%), specificity (+ 13%), mcc (+ 0.1) and g-means value (+ 5.1). Evaluations using tenfold cv and training/testing splits confirm the competitive performance.

**Conclusions:**

A comparative analysis was performed to assess the effect of three different sampling methods (random, *K*-means and uniform sampling) on the training of learning algorithms using different evaluation methods. Models trained on *K*-means sampled datasets generally show a significantly improved performance compared to those trained on random selections—with RF seemingly benefiting most in our particular setting. Our findings on the sampling are highly relevant and apply to many applications of supervised learning approaches in bioinformatics.

## Background

Insights into protein–protein interactions (PPIs) can shed light into the molecular mechanisms of biological processes, inform about pathogenesis, and help identify disease intervention points. The prevalent experimental methods include the two-hybrid system [1, 2] and co-immunoprecipitation [3, 4], which are both used for large scale determination of PPIs. Computational methods for PPI prediction can complement experimental methods as they are cost effective and less time consuming [5]. Aside from general PPI predictors, computational approaches have also been developed for specific subsets of proteins, e.g. those encoded by paralogous genes [6, 7]. Among the different computational methods, Machine Learning (ML) based prediction methods provide a suitable alternative to experimental approaches, allowing for near accurate and fast annotation of biological sequences. These methods exploit hidden similarities to known interacting protein pairs based on various calculated protein features, including sequence, physicochemical, evolutionary, and structural information [8]. Usually, machine learning predictors that are based on a diverse feature set achieve higher classification accuracy than traditional, solely sequence similarity based methods [9, 10]. Several ML systems for PPI prediction have been developed, using a variety of learning methods and feature sets [5, 11–18]. A specific case of a PPI is a cytokine–receptor interaction (CRI). Cytokines are a rather loosely defined group of small signaling proteins that bind specific receptors on the plasma membrane of target cells. Knowledge of CRIs is very important for understanding the pathogenesis of various human diseases—notably autoimmune, inflammatory and infectious diseases—as well as for identifying potential therapeutic targets. While computational prediction of CRIs can narrow down the search space for wet lab based experimental validation screens, only few computational studies have specifically contributed towards the prediction of cytokines and CRIs [19–23].

Previously, Wei et al. [24] used k-skip-gram, physicochemical properties and local pseudo position-specific scoring information with a random forest based classifier for developing a prediction model. Their method achieved an overall accuracy of 83.7% with 80.8% sensitivity and 86.7% specificity. Since the specificity is higher than the sensitivity, it can be concluded that their prediction model is more accurate for predicting non-interacting pairs than interacting pairs. Wei et al. [22] further improved the CRI prediction accuracy using random forest with evolutionary features, i.e. Pseudo Position-Specific Score Matrix (Pse-PSSM) and amino acid composition and PSSM with auto-covariance (AC) transformation (AAC_PSSM_AC). Using leave one out cross validation (loocv), they obtained an overall accuracy of 87.9%, a sensitivity of 92.6% and a specificity of 83.3%, leaving room for further improving CRI predictions.

Both positive examples (interacting pairs) and negative examples (non-interacting pairs) contribute towards the optimal learning of a classifier. Positive datasets are easy to obtain from a number of databases. However, “Gold standard” negative datasets, which could be used for benchmarking, are still lacking. There is no agreement on the method for the creation of a “standard” dataset of non-interacting proteins [25]. As a result, a variety of methods for creating high quality non-interacting protein partners have been proposed. One such method is to select protein pairs with different (annotated) sub-cellular localizations, because they are unlikely to interact [26, 27]. Probably the simplest method reported for forming a set of non-interacting protein pairs is to sample from the pool of all those pairs that are not known to interact, i.e. combinations absent from the PPI list [11, 28, 29]. Another method is to use structural similarity as a criterion [30]. The expectation is that if a pair of proteins is structurally very similar to another pair of proteins that are known to interact, then these two proteins are likely interacting as well. However, the above mentioned methods to produce high quality negative datasets have their pitfalls: Sampling based on non-colocalization of protein pairs may lead to over-optimistic performance evaluation metrics for the ML classifier, as it is easier to discriminate these negative examples, since they are biased with localization information [31]. Random sampling of protein pairs not present in the protein interaction list may result in a negative dataset containing putative interacting protein pairs, even though this has been estimated to occur only with very low probability [25]. And significant structural similarity can also exist among non-interacting pairs and interacting pairs. For instance, approximately 8.7% of non-interacting pairs in *S. cerevisiae* are expected to be structurally similar to interacting pairs [32]. Of note, it is important to distinguish between the underlying principle used to generate the base set of non-interacting protein pairs (e.g. sub-cellular non-co-localization, structural dissimilarity, PPI set exclusion) and the actual methods used to sample from this base set for the purpose of generating a much smaller negative dataset that the ML models are being trained on.

We evaluated a number of different ML algorithms with simple sequence-based features along with evolutionary information. In order to enhance the prediction accuracy further, we developed a heterogeneous feature set based on individual feature performances with respect to the different learning algorithms. The heterogeneous feature set consisting of atomic composition, physicochemical-2-grams, AAC_PSSM and D-FPSSM produced the best performance evaluation metrics based on loocv.

Anomaly detection can be defined as the problem of finding patterns in the data that do not fit to the expected behavior or normal behavior. These “not fitting patterns” are called by a variety of names such as anomalies, aberrations, or exceptions [33]. A plethora of methods such as statistical profiling using histograms, neural networks, mixture models, support vector machines, and clustering have been used successfully for anomaly detection [33]. In the present work we use the anomaly detection capability of autoencoders for constructing different sets of negative samples and show how different categories of negative samples can affect the training of a learning algorithm.

Strong biases in negative datasets can significantly affect the training of learning algorithms and their evaluation. Imbalanced datasets having a high skew towards negative samples result in majority class classifiers [21, 34]. Consequently, unbiased negative sets of non-interacting proteins are desirable. We propose a method for negative data subset sampling using *K*-means clustering, which enhances the learning and evaluation of the entire input space by providing a proper and diverse representation. A representative training set has a proper distribution of samples from the entire input space, which is a principal requirement for near complete learning. Likewise, a properly diversified test set is also needed for robust evaluation of the learned models. To this end, we thoroughly evaluated all models using different sampling and performance evaluation methods. The latter include (1) loocv, (2) tenfold cross validation, (3) random and diversified (*K*-means based) training/testing, and (4) uniform sampling-based training/testing sets. Our results show that negative dataset selection and sampling have significant impact on prediction accuracy of ML models, and lead to an improved performance of CRI predictors. A schematic representation of our approach is shown in Additional file 1: Fig. S1.

## Results and discussion

Each of the selected ML algorithms was applied using the original dataset by Wei et al. [22] and various feature sets (see “Methods” below and Additional file 1). The performance was evaluated based on loocv (Additional file 1: Tables S1–S12 and Table 1). With the feature set changing and depending on the performance metric, the best performing algorithm changes. For AAC, DPC, PGC and PCP feature sets, the accuracies for the ML algorithms range between 63.1% and 81.0% (Additional file 1: Tables S1, S2, S3 and S4). The maximum accuracy is achieved by the SMO-RBF model trained using AAC features. This combination also achieves the highest accuracy, mcc and g-means, while the best AUC value is achieved with the RF model trained using DPC features. Using either P2G or ATC feature sets (Additional file 1: Tables S5, S6) generally improves the performance of all learning algorithms, in particular the accuracy of correctly predicting CRIs (higher sensitivity). All ML algorithms achieve more than 80% sensitivity using either of these two feature sets, while higher sensitivities are achieved with the ATC feature set. More specifically, the RF model using ATC features performs best in all metrics except sensitivity, where the SMO-RBF model using ATC achieves the highest value (94.6%), and AUC, where the RF model using P2G achieves the highest value (0.904). Using the combined feature sets of ATC and P2G (Additional file 1: Table S7), the obtained sensitivities are well above 80% for all algorithms. However, for IBK and RF the sensitivity is slightly decreased as compared to the scenario where only the ATC feature set is used. Overall, the combination of ATC and P2G features improves performances of only a few models (such as bagging) but other algorithms do not benefit from the larger feature set. Of note, the AUC for the RF-based model is slightly higher (0.912).

**Table 1:** Performance metrics of different ML algorithms using the combined features ATC + P2G + AAC_PSSM + D-FPSSM when applied to *exactly the same dataset (positives and sampled negatives) used by *Wei et al. [22]

	SE	SP	ACC	MCC	AUC	g-means
ATC + P2G + AAC_PSSM + D-FPSSM						
NB	94.1	68.5	81.3	0.647	0.843	80.2
A1DE	*95.1*	69.5	82.3	0.668	0.910	81.2
SMO-RBF	93.6	86.7	*90.1*	*0.805*	0.901	*90.1*
SMO-PolyK	92.6	77.3	85.0	0.708	0.850	84.6
SMO-PuK	90.6	*88.2*	89.4	0.788	0.894	89.4
IBK	91.6	84.2	87.9	0.761	0.879	87.8
Bagging	85.7	79.8	82.8	0.656	0.904	82.7
RF	89.2	81.3	85.2	0.707	*0.935*	85.1
Wei et al	92.6	83.3	87.9	0.762	–	87.8

For comparison, we also show the metrics reported by Wei et al. The best performance for each metric is shown in italic

Most learning algorithms perform much better using the AAC_PSSM or the D-FPSSM feature set than when using only sequence-based feature sets (Additional file 1: Tables S8 and S9, respectively). Except for NB (for AAC_PSSM and D-FPSSM) and IBK (for D-FPSSM), all other algorithms produced trained models with more than 80% accuracy. The highest accuracy (87.2%) and specificity (88.2%) is obtained using SMO-PuK with AAC_PSSM, the highest AUC value is achieved using an RF model with D-FPSSM (0.939), and NB with D-FPSSM yields the highest sensitivity (97.5%). With minor exceptions, combining the two feature sets increases specificities compared to models trained with D-FPSSM features, and increases sensitivities compared to models trained with AAC_PSSM features (Additional file 1: Table S10). Notably, SMO-RBF benefits from training using both feature sets, as the model achieves the highest accuracy (89.2%). Overall, AAC_PSSM and D-FPSSM features and their combination performed better than all other sequence-based features (Additional file 1: Table S12).

Training the different ML algorithms using the full feature set let to the following results (Additional file 1: Table S11): SMO-RBF achieved the highest recorded specificity (99.5%) over all algorithms and feature configurations, but its sensitivity was significantly lower in comparison to all other ML algorithms. The highest accuracy and sensitivity are obtained by A1DE but does not reach the values of the best performing models trained using AAC_PSSM and/or D-FPSSM.

Based on the performance of the ML algorithms on the different feature sets, we decided to merge those sets that had trained models with more than 80% accuracy. This resulted in a new feature set created by the combination of ATC, P2G, AAC_PSSM and D-FPSSM features (the length of the vector describing the features of one protein is equal to 5 + 11 + 20 + 20 = 56). Using this feature set, we obtained a SMO-RBF model with 90.1% accuracy, an mcc value of 0.805, and a g-means value of 90.1—the highest values over all algorithms and feature configurations (see Table 1). Moreover, the model was 2.2% more accurate than the model previously described by Wei et al. [22], which had been trained on the same dataset. The highest AUC value among all trained models with this particular feature set was obtained with the RF model (0.935). Of note, we achieved a higher accuracy with a smaller feature set, which has the additional benefit of a shorter classifier training time. Our feature vector had only 112 (2 × 56) dimensions as compared to 1600 dimensions of the feature vectors used in [22].

Based on these performance metrics, we chose SMO-RBF for investigating further the effect of sampling non-interacting pairs on the training and the performance of our classifiers. For that purpose, we used the anomaly detection capability of deep autoencoders and explored some of the parameters for selecting the best autoencoder model, evaluated in terms of MSE and RMSE. For all autoencoder models we fixed the number of epochs to 1000, the l2 regularization parameter to 0.0001, and experimented with different architectures. Specifically, we used either decreasing, increasing or alternating larger and smaller numbers of neurons with varying numbers of hidden layers, i.e. we varied both width and depth of the autoencoder models. The MSE and the RMSE of the autoencoder model with rectifier activation function was very high (model no. 2, Additional file 1: Table S13), and, hence, we decided to use *tanh* as the activation function for all other autoencoder models. The best autoencoder model achieved an MSE of 0.00067 and an RMSE of 0.0258 (model no. 5, Additional file 1: Table S13) using three hidden layers with decreasing numbers of neurons: 1000, 500, and 100.

The unsupervised autoencoder was trained with only positive samples i.e. interacting protein pairs. However, both the positive samples and the negative samples were passed through the autoencoder for the estimation of the reconstruction error. The respective histogram bins and the counts for the reconstruction error of interacting protein pairs and non-interacting protein pairs are presented in Additional file 1: Fig. S2 and Table S14 (Additional file 1). Almost all the interacting protein pairs have a reconstruction value ≤ 0.003, which is intuitively understandable since the autoencoder was trained on this dataset. Moreover, the non-interacting protein pairs with lower reconstruction error are closer to the interacting protein pairs (see also additional information in the Additional file 1).

In order to group the (potentially) negative samples according to how difficult it is to classify them correctly, namely into “easy to classify” non-interacting pairs, “difficult to classify” non-interacting pairs, and those that are in-between these two groups, we divided the negative samples according to their estimated autoencoder reconstruction error. According to our data, 0.003 was a reasonably good cut-off to distinguish between the “difficult to classify” positive pairs and the rest, while 0.001 was a reasonably good cut-off to distinguish the “easy to classify” positive pairs from the rest. We then used the same cutoff to group the “easy to classify” and the “hard to classify” non-interacting proteins. Accordingly, we obtained the following three groups: (a) samples with reconstruction error ≤ 0.001, (b) those with reconstruction error > 0.001 and ≤ 0.003, and (c) samples with reconstruction error > 0.003. Subsequently, we trained SMO-RBF models using all the 203 positive samples and 203 negative samples, all randomly selected from one of the above three classes. The performance metrics of five SMO-RBF models and their average, evaluated using loocv for each of the three classes, are shown in Additional file 1: Table S15. As expected from obtained reconstruction errors and confirmed by the reported specificity and accuracy of SMO-RBF models, the negative samples belonging to the first group (≤ 0.001) are hardest to classify, due to the closeness of the non-interacting pairs to interacting pairs creating overlapping decision boundaries, while those belonging to the third group (> 0.003) are easiest to classify.

We concluded, using random sampling for selecting instances for the negative training data does not guarantee a wide representation of the entire negative sample space as there is always a chance of either overrepresentation or underrepresentation of, for instance, hard or easy to classify instances. With respect to the above mentioned three groups, if the set of negative instances in the training set consists mostly of samples belonging to group (c), then the learning algorithm will most likely return overoptimistic performance evaluation metrics. To mitigate the under-representativeness or over-representativeness of the random selection strategy we decided to use *K*-means sampling (with *K* = 203, the number of positive instances) for constructing representative negative training sets (see “Methods” section). The distribution of samples into the different clusters are shown in Additional file 1: Fig. S3 for all the negative samples.

In Tables 2, 3 and 4 we present the performance metrics for all eight learning algorithms using loocv (Table 2), random training (70%) and testing (30%) split (Table 3), and tenfold cv (Table 4). Results are averaged over ten randomly (Tables 2, 3 and 4, top) and ten *K*-means (*K* = 203) sampled negative datasets (Tables 2, 3 and 4, bottom). Our results obtained using loocv with randomly sampled negatives (Table 2, top) show that SMO-PuK performs best for all metrics (SP: 83.5%, ACC: 78.2%, AUC: 0.782, mcc: 0.568, g-means: 78.0) except for sensitivity. Here the ‘winner’ is IBK (81.4%). SMO-RBF is the second-best performing algorithm for all metrics (SP: 74.5%, ACC: 76.7%, AUC: 0.767, mcc: 0.536, g-means: 76.7). Also, IBK performs similarly to SMO-RBF for those metrics. Performances of all algorithms improve significantly when training is done with sets of negatives obtained via *K*-means based sampling (see Table 2, bottom). In this case, RF becomes the best performing algorithm for all metrics except sensitivity (SP: 96.3%, ACC: 93%, AUC: 0.965, mcc: 0.862, g-means: 92.9). SMO-RBF performs best with respect to sensitivity (SE: 90.2%). It also performs second-best for metrics accuracy (90.5%) and g-means (90.5). Of note, all other algorithms including NB, A1DE, IBK, and Bagging also perform much better when trained with negatives obtained via *K*-means sampling than when trained on randomly sampled negatives, indicating the significant impact that the negative training data has on the performance of all learning algorithms. In accordance with the results in Table 2, (almost) all performance metrics increase considerably (for all ML algorithms) on the holdout disjunct testing set, when the training is performed using *K*-means sampled diversified training sets (Table 3, bottom) instead of random sampling (Table 3, top). NB’s specificity is the only exception. As to random sampling, SMO-PuK and SMO-RBF perform best considering all metrics. While IBK performs best in terms of sensitivity (79.8%), and RF performs best in terms of AUC (0.779), SMO-PuK performs best on all other metrics closely followed by SMO-RBF. When *K*-means sampling is used, performances improve most significantly for RF, that performs now best for all metrics (SE: 90.3%, SP: 93.9%, ACC: 92.2%, mcc: 0.844, AUC: 0.959, g-means: 92.0). SMO-RBF performs second-best in a number of metrics (SE: 89.2%, ACC: 89.2%, mcc: 0.785, and g-means: 89.2). When applying 10 × 10-fold cv (see Table 4), the performances and, thus, the ranking of the ML algorithms is very similar to those reported for Tables 2 and 3. Lastly, we also applied uniform sampling (Kennard Stone algorithm), both to obtain an initial sampling of 203 negative instances and in combination with the training/testing split. In general, performances were better compared to random sampling but not as good as when using *K*-means based sampling (see Additional file 1: Tables S16 and S17 and additional information in the Additional file 1).

**Table 2:** Performance metrics of different ML algorithms using negative samples generated through random (top) and *K*-means (bottom) sampling

	SE	SP	ACC	MCC	AUC	g-means
Random (loocv)						
NB	52.3	59.3	55.8	0.119	0.554	54.9
A1DE	70.5	53.3	62.9	0.264	0.707	62.5
SMO-RBF	79.1	74.5	76.7	0.536	0.767	76.7
SMO-PolyK	62.8	50.9	56.9	0.199	0.569	56.4
SMO-PuK	73.0	*83.5*	*78.2*	*0.568*	0.782	*78.0*
IBK	*81.4*	71.9	76.6	0.536	0.766	76.4
Bagging	66.6	63.5	65.1	0.303	0.718	65.0
RF	73.2	71.6	72.4	0.449	*0.807*	72.3
*K*-means (loocv)						
NB	73.3	51.3	61.3	0.253	0.713	63.0
A1DE	83.6	84.1	83.9	0.679	0.908	83.8
SMO-RBF	*90.2*	90.8	90.5	0.814	0.905	90.5
SMO-PolyK	80.5	87.9	84.2	0.686	0.842	84.0
SMO-PuK	85.7	91.7	88.4	0.770	0.887	88.3
IBK	86.1	86.1	86.1	0.722	0.861	86.0
Bagging	86.4	92.3	89.4	0.789	0.945	89.3
RF	89.7	*96.3*	*93.0*	*0.862*	*0.965*	*92.9*

The averages of ten runs of *loocv* are reported. The best performance for each metric is shown in italic

**Table 3:** Average performance metrics of different ML algorithms based on 10 runs of *split training and testing sets*

	SE	SP	ACC	MCC	AUC	g-means
Random (70% training, 30% testing)						
NB	63.7	53.5	58.4	0.178	0.595	56.8
A1DE	70.7	57.5	62.2	0.255	0.674	63.4
SMO-RBF	79.3	72.5	75.7	0.520	0.759	75.6
SMO-PolyK	66.7	50.0	55.2	0.194	0.583	57.1
SMO-PuK	74.8	*77.8*	*76.3*	*0.529*	0.763	*76.0*
IBK	*79.8*	68.9	74.1	0.491	0.743	73.9
Bagging	65.5	58.9	62.0	0.244	0.682	62.0
RF	74.5	64.2	69.1	0.390	*0.779*	68.9
*K*-means (70% training, 30% testing)						
NB	79.7	50.0	63.6	0.299	0.742	62.3
A1DE	77.6	87.2	82.9	0.659	0.857	82.4
SMO-RBF	89.5	89.0	89.2	0.785	0.890	89.2
SMO-PolyK	88.1	78.9	83.2	0.692	0.828	83.2
SMO-PuK	82.9	85.1	84.1	0.683	0.845	83.8
IBK	82.2	87.0	84.2	0.692	0.841	84.0
Bagging	84.8	92.3	88.7	0.776	0.934	88.4
RF	*90.3*	*93.9*	*92.2*	*0.844*	*0.959*	*92.0*

The different sets are generated through either random (top) or *K*-means sampling (bottom). As for the latter, each run, the 12,343 negative protein-receptor combinations undergo *K*-means sampling with *K* = 203. The randomly chosen 203 negative samples, one per cluster, are then randomly split into training (70%) and testing (30%) sets. Similarly, the 203 positive samples are randomly split into training (70%) and testing (30%) sets

**Table 4:** Performance metrics of different ML algorithms averaged over *10 × 10-fold cross validations*

	SE	SP	ACC	MCC	AUC	g-means
Random (10 × 10-fold cross validation)						
NB	51.04	61.22	55.49	0.106	0.560	66.2
A1DE	72.02	52.4	62.2	0.244	0.697	61.4
SMO-RBF	79.70	75.57	77.57	0.553	0.776	*77.6*
SMO-PolyK	64.72	49.45	57.11	0.144	0.571	56.5
SMO-PuK	71.76	*83.29*	*78.53*	*0.555*	0.775	77.3
IBK	*81.14*	71.43	76.28	0.528	0.762	76.1
Bagging	66.65	63.42	65.02	0.301	0.712	65.0
RF	72.77	69.79	71.27	0.426	*0.795*	71.2
*K*-means (10 × 10-fold cross validation)						
NB	74.05	51.76	61.76	0.262	0.714	61.9
A1DE	83.70	85.02	84.34	0.688	0.901	84.4
SMO-RBF	88.55	90.48	89.53	0.796	0.903	89.5
SMO-PolyK	82.72	88.06	85.39	0.709	0.847	85.3
SMO-PuK	84.60	91.32	87.97	0.761	0.880	87.7
IBK	84.42	86.35	85.4	0.711	0.855	85.4
Bagging	86.10	92.25	89.19	0.786	0.938	89.1
RF	*89.42*	*95.98*	*92.52*	*0.853*	*0.957*	*92.6*

Each model was trained on different negative sample sets that have been generated using either random selection or the *K*-means based sampling

## Conclusions

Knowledge of CRIs can facilitate the understanding of many regulatory biological processes. ML-based CRI prediction can help narrowing down the search space for wet lab based experimental validation. In the present work, we tested a number of different feature sets. The feature set consisting of atomic composition, physicochemical-2-grams as well as evolutionary information in the form of AAC_PSSM and D-FPSSM proved to be very useful in developing CRI predictors. To the best of our knowledge, this study is the first in using atomic composition and physicochemical-2-grams as discriminating features for PPI. We then compared the effect of random, uniform and *K*-means based sampling of the negative data on the training of eight different machine learning algorithms. To this end, we produced 10 different negative sample sets for each method. The trained ML models were thoroughly evaluated using the popular leave one out cross validation, 10 × 10-fold cross validation, and 70% training/30% testing split. Our results show, performances of all ML algorithms benefitted from *K*-means based sampling, with RF models performing best, significantly outperforming the Wei et al. [22] model with respect to all metrics except sensitivity. Negative dataset selection as well as sampling have significant impact on prediction performance. *K*-means sampling with random splitting produced diversified training sets consisting of representative samples that cover the input space and include both common and rare patterns, thus allowing for a complete learning of the ML algorithms. Corresponding diversified testing sets led to a proper and robust evaluation of the trained models, showing a considerable increase of their generalization ability. So far, a gold standard for choosing non-interacting protein pairs has been lacking. Here we have shown that *K*-means based sampling is able to include samples from the entire representative list of protein non-interactions, thereby avoiding underrepresentation or overrepresentation and learning bias. The good performance metrics of the trained RF models on *K*-means sampled datasets across all evaluation methods suggest that the present methodology can be used as a complementary approach along with other wet lab methods for CRI identification.

## Methods

### Dataset

We use the original dataset of Wei et al. [22] in order to allow for a direct comparison. It consists of 123 human cytokines and 102 human receptors. Thus, there are 12,546 (= 123 × 102) possible cytokine–receptor combinations. Among those are 203 known interacting cytokine–receptor pairs that form the positive training dataset (see Additional file 1). As per the “closed world association” [14], all cytokine–receptor pairs not present in the positive dataset are potentially negative. This pool of protein pairs that are not known to interact comprises in total 12,343 pairs. The negative training data in [22] was obtained from this set by simple random sampling of 203 pairs. Aside from training ML models with the exact same negative data in [22], we also generated new negative training data by sampling 203 non-interacting cytokine–receptor pairs from the same pool of protein pairs (accepting the relatively small risk of having false negatives in this pool). For the work reported here, we used three different sampling methods: simple random sampling as in [22], *K*-means clustering and sampling, and uniform selection algorithm for sampling. The latter two are described below in the subsection “Sampling Methods for Creating Diversified and Representative Training and Testing Sets “.

### Feature extraction

The prediction of protein classifications (interacting/non-interacting, family classification, etc.) is profoundly dependent on feature extraction. Data representation is an important part in the training of ML algorithms, because different representations can emphasize different aspects of the data. The accuracy of the learned model is strongly impacted by the selection of features that we feed into the learning phase of the classifier. Broadly speaking, two types of features are calculated for distinguishing interacting pairs from non-interacting pairs: (1) Classical sequence-based features and (2) features based on evolutionary information. From the first feature type we use Amino Acid Composition (AAC), Dipeptide Composition (DPC), Property Group Composition (PGC), Physicochemical-2grams (P2G), Atomic Composition (ATC), and Physicochemical Properties (PCP). Regarding the second feature type, we use two types of information representations from PSSMs (position specific scoring matrices), namely AAC_PSSM and D-FPSSM. For performance evaluation of different ML algorithms, we use each of these features individually and in addition the following combinations of feature sets: ATC + P2G, AAC_PSSM + D-FPSSM, ATC + P2G + AAC_PSSM + D-FPSSM, and all features combined. A detailed description of each individual feature is provided in the Additional file.

### Classification algorithms

We have compared eight different machine learning algorithms: Naïve Bayes (NB), Average One Dependence Estimator (A1DE), Support vector machines with RBF kernel (SMO-RBF), Support vector machines with Poly K kernel (SMO-Poly K), Support vector machines with PuK kernel (SMO-PuK), Nearest neighbor classifier (IBK), Bagging, and Random Forest (RF). The description of each of the algorithms is provided in the Additional file 1.

We have implemented all ML algorithms on the Weka ML platform [35] using default parameters. Of note, our results (including the best performing classifier) could have varied greatly had we applied parameter optimization methods.

### Autoencoders

An autoencoder is an artificial neural network used for unsupervised learning. Its main objective is to learn hidden structures from unlabeled data by attempting to reproduce the input given to it through its hidden layer representations [36, 37] (Additional file 1: Fig. S1). It consists of simulated layers of neurons, where each neuron is associated with a weight and an activation function. The simplest autoencoder consists of an input layer, an output layer where the reconstructed input is produced, and a single hidden layer between those two. So-called “deep” autoencoders have more than one hidden layer. In traditional neural networks, the effect of backpropagation decreases on the first layer with increasing number of hidden layers, while in deep learning neural networks, each layer is trained independently and the feature encodings from the previous layer are used to train the successive layers. Deep learning neural networks overcome the vanishing gradient problem known to occur in gradient based learning of traditional neural networks with multiple layers. In deep autoencoders the output of the preceding layer becomes the input of the successive layer. The layout of the autoencoder (i.e. its architecture), including the number of hidden layers, the type of activation function and the number of epochs it has to be trained, and the choice of regularization parameters such as l2 (also known as ridge regression) are prespecified. The goal is to achieve a model with minimum error between the actual input X_i_ and the reconstructed input O_i_. Let *n* be the number of features used to represent the data. Then the reconstruction error ∆i is computed as

$$\Delta i = \frac{1}{n}\mathop \sum \limits_{j = 1}^{n} \left( {X_{i,j} - O_{i,j} } \right)^{2} .$$

Autoencoders find application in unsupervised pre-training of deep learning neural networks and in anomaly detection [38]. In the present work we used autoencoders for negative dataset creation using its anomaly detection capability. We trained different autoencoder models with the positive interacting pairs only. The best trained autoencoder model (i.e. with minimum mean squared error and root mean squared error) is then used to categorize all the negative interacting pairs using the calculated reconstruction error values. A non-interacting pair having proximity to interacting pairs will have low reconstruction error. Conversely, non-interacting pairs with high reconstruction error are very dissimilar to interacting pairs. Based on the values of the reconstruction error, we categorized the non-interacting pairs into different groups for the training of a classifier. We used the H2O package (https://h2o.ai) in R for implementing the autoencoder models.

### Performance metrics

We have evaluated the ML models using a wide range of performance metrics, using both threshold-dependent and threshold-independent parameters. Let *TP*, *TN*, *FP*, and *FN* denote the number of correctly predicted CRI pairs (true positives), the number of correctly predicted non-interacting pairs (true negatives), the number of non-interacting pairs predicted incorrectly as cytokine–receptor interacting pairs (false positives) and the number of CRI pairs predicted incorrectly as non-interacting pairs (false negatives), respectively. Our metrics are:

*Sensitivity*: Percentage of correctly predicted CRI pairs:

$$SE = 100 \times TP/\left( {TP + FN} \right).$$

*Specificity*: Percentage of correctly predicted non-interacting pairs:

$$SP = 100 \times TN/\left( {TN + FP} \right).$$

*Accuracy*: This metric expresses the correctly predicted cytokine–receptor and non-interacting pairs.

$$ACC = \left( {TP + TN} \right) \times 100/\left( {TP + FP + TN + FN} \right).$$

*Mathews correlation coefficient (MCC)*: It is a valuable measure for binary classification problems. Values range from − 1 to + 1.

$$MCC = \frac{{\left( {TP \times TN} \right) - \left( {FP \times FN} \right)}}{{\sqrt {\left( {TP + FN} \right)\left( {TP + FP} \right)\left( {TN + FP} \right)\left( {TN + FN} \right)} }}$$

*Area under ROC (AUC):* The AUC can be used to summarize ROC (receiver operating characteristic) curves by a single numerical quantity. Possible values of the AUC range from 0 to 1. The closer the value to 1 the better the performance of the ML model [19, 39, 40].

*g-means*: This is the geometric mean of sensitivity and specificity and is calculated by the following formula [41]:

$$g{\text{-}}means = \sqrt {SE \times SP} .$$

It gives a balanced view about the performance of the ML models for both the positive and negative classes.

For the evaluation of autoencoder models, we have used the following performance evaluation metrics:

*Root mean squared error (RMSE)*: Also known as the root mean square deviation, the RMSE is calculated as the difference between the values predicted by a model and the values actually observed from the real environment that is being modeled:

$$RMSE = \sqrt {\mathop \sum \limits_{i = 1}^{n} \left( {X_{obs,i} - X_{model,i} } \right)^{2} /n} ,$$

where *n* is the number of samples, *X*_*obs,i*_ is the observed value and *X*_*model,i*_ is the predicted value.

*Mean squared error (MSE)*:

$$MSE = \frac{1}{n}\mathop \sum \limits_{i = 1}^{n} \left( {X_{obs,i} - X_{model,i} } \right)^{2} .$$

### Performance evaluation

Three different evaluation methods were used to infer the generalization ability and the robustness of the ML models. (1) Leave one out cross validation (loocv). loocv is a special case of K-fold cross validation, where K is equal to the number of samples. In each round of evaluation, one sample is left out as a test case and all the other samples are used to train the model. This process is repeated until all the samples are evaluated once as a test case. Loocv is computationally expensive, but we decided to include this evaluation method since it had been used previously to evaluate the models in the work of Wei et al. [22], to which we are comparing our models. (2) 10 × 10-fold cv (tenfold cv repeated 10 times), (3) Random training and testing. Here, a fixed percentage of data is kept aside for training and the rest of the data is used for testing the model. The training and testing sets are mutually exclusive. Random splitting of training and testing sets leaves room for over-representation or under-representation of patterns that belong to different classes in training and testing sets. As a consequence, it can give variable estimates for the prediction error. If the training set lacks a subset of patterns, the learning algorithm will be unable to identify that subset of patterns. Likewise, if a subset of patterns is missing in the testing set, then this leads to an incomplete evaluation of the ML model. In other words, if the training and testing sets are created using random sampling, then there is no guarantee for the inclusion of both common and rare patterns in the training set. To overcome these shortcomings of incomplete learning and unrepresentativeness, we implemented a diversified training/testing set.

### Sampling methods for creating diversified and representative training and testing sets

A diversified training set has proper representative samples from the entire input space and is a necessary prerequisite for complete learning [8, 42]. Similarly, a diversified testing set is needed for the true estimation of the classification error of the trained models. Ideally, a training set is free from both between and within class imbalance. In the present case, the numbers of samples of interacting and non-interacting proteins are equal, so there is no between class imbalance. Within class imbalance arises due to the difference in the number of common and rare patterns belonging to each class. These rare patterns are also known as small disjuncts [43].

Applied to our problem this means that if the negative samples in the training set represent only a relatively narrow sample space, then this can bias the learning and result in over-optimistic or over-pessimistic performance evaluation metrics. Consequently, a sampling strategy that produces a set of negative instances that effectively represents (as much as possible) the entire negative sample space would be highly advantageous for the training of ML models. Such trained models would have the opportunity to learn from instances belonging to many different regions of the entire negative instance space.

For the purpose of creating diversified and representative training and testing sets, we used the unsupervised *K*-means clustering algorithm. The aim of *K*-means clustering is to partition the variety of patterns (samples) into a predefined number of clusters (*K*) based on the similarity/dissimilarity that exists among the patterns (samples) [8]. Ideally, the similarity between the clusters is at a minimum, while the similarity within the clusters is at a maximum. *K*-means clustering proceeds by minimizing the objective function given by

$$SSE = \mathop \sum \limits_{j = 1}^{K} \mathop \sum \limits_{i = 1}^{{n_{j} }} P_{i}^{j} - C_{j} ,$$

where *SSE* is the acronym for “sum of squared errors”, $C_{j}$ denotes the centroid of the *j*th cluster, $P_{i}^{j}$ denotes the *i*th pattern of the *j*th cluster, *n*_*j*_ denotes the number of objects in the *j*th cluster, *K* denotes the predetermined number of clusters, and **||**·**||** denotes the Euclidian distance.

All input features are normalized prior to the application of the *K*-means algorithm. Specifically, all the numerical values (attribute values) are normalized in the range from 0 to 1 using the min–max normalization method [44]. We use *K* = 203 (i.e. equal to the number of positive interacting pairs) for clustering the 12,343 non-interacting pairs. Then, for generating a *K*-means sampled negative dataset, we select one instance from each non-interacting pair cluster for the training set. Thus, our final combined training and testing set consisted again of 203 negative instances (one from each cluster) and of all 203 positive instances.

Of note, *K*-means sampling could potentially favor the selection of false negatives if present in the negative base set. This is so because the selection for broad representativeness favors the selection of the most dissimilar feature vectors, which are most suspicious of being false negatives (i.e. it is assumed that false negatives are likely to be found among the most dissimilar negatives). However, the likelihood of having false negatives is still relatively small, perhaps even smaller than the likelihood of having false positives [25].

Furthermore, we have implemented the uniform selection algorithm for sampling. Here, the representative samples are selected with the aim of uniformly covering the entire input space. The uniform selection algorithm, also known as Kennard-Stone algorithm, is implemented as described in [45]. The algorithm proceeds by selecting the sample that is closest to the data mean, which is then added to the representative set. Using Euclidean distance as the dissimilarity metric, new samples are iteratively selected among those that are most dissimilar to the samples already present in the representative set.

## Supplementary information

**Additional file 1**. Supplementary material (docx file).

**Additional file 2**. 10 times 10-fold CV test/train sets in ARFF format (zip file).

**Additional file 3**. 10 K-means sampled test/train sets in ARFF format (zip file).

## Data Availability

The sequence IDs are provided in the Additional file. Random and *K*-means sampled data is provided in ARFF format (see Additional files list).

## Abbreviations

CRI: Cytokine receptor interaction
RF: Random forest
loocv: Leave one out cross validation
PPIs: Protein–protein interaction
ML: Machine learning
Pse-PSSM: Pseudo position-specific score matrix
AAC: Amino acid composition
DPC: Dipeptide composition
PGC: Property group composition
P2G: Physicochemical-2grams
ATC: Atomic composition
PCP: Physicochemical properties
MSE: Mean squared error
RMSE: Root mean squared error
RE: Reconstruction error
NB: Naive Bayes
IBK: K-nearest neighbor
A1DE: Average one dependence estimator
SMO-RBF: Sequential minimization optimization-radial basis function
MCC: Mathews correlation coefficient
ACC: Accuracy
SP: Specificity
SE: Sensitivity
ROC: Receiver operating characteristic
AUC: Area under ROC curve

## References

[1] Cagney G, Uetz P, Fields S, Thorner J, Emr SD, Abelson JN (2000). High-throughput screening for protein–protein interactions using two-hybrid assay. Methods in enzymology.

[2] Uetz P, Hughes RE (2000). Systematic and large-scale two-hybrid screens. Curr Opin Microbiol.

[3] Gavin A-C, Aloy P, Grandi P, Krause R, Boesche M, Marzioch M, Rau C, Jensen LJ, Bastuck S, Dümpelfeld B (2006). Proteome survey reveals modularity of the yeast cell machinery. Nature.

[4] Gavin A-C, Bösche M, Krause R, Grandi P, Marzioch M, Bauer A, Schultz J, Rick JM, Michon A-M, Cruciat C-M (2002). Functional organization of the yeast proteome by systematic analysis of protein complexes. Nature.

[5] Zahiri J, Bozorgmehr JH, Masoudi-Nejad A (2013). Computational prediction of protein–protein interaction networks: algorithms and resources. Curr Genomics.

[6] Bitbol A-F (2018). Inferring interaction partners from protein sequences using mutual information. PLoS Comput Biol.

[7] Gueudré T, Baldassi C, Zamparo M, Weigt M, Pagnani A (2016). Simultaneous identification of specifically interacting paralogs and interprotein contacts by direct coupling analysis. Proc Natl Acad Sci.

[8] Nath A, Subbiah K (2017). The role of pertinently diversified and balanced training as well as testing data sets in achieving the true performance of classifiers in predicting the antifreeze proteins. Neurocomputing.

[9] Nath A, Subbiah K (2015). Maximizing lipocalin prediction through balanced and diversified training set and decision fusion. Comput Biol Chem.

[10] Ramana J, Gupta D (2009). LipocalinPred: a SVM-based method for prediction of lipocalins. BMC Bioinform.

[11] Gomez SM, Noble WS, Rzhetsky A (2003). Learning to predict protein–protein interactions from protein sequences. Bioinformatics.

[12] Lei Y, Jun-Feng X, Jie G (2010). Prediction of protein–protein interactions from protein sequence using local descriptors. Protein Pept Lett.

[13] Martin S, Roe D, Faulon J-L (2005). Predicting protein–protein interactions using signature products. Bioinformatics.

[14] Roy S, Martinez D, Platero H, Lane T, Werner-Washburne M (2009). Exploiting amino acid composition for predicting protein–protein interactions. PLoS ONE.

[15] Sprinzak E, Margalit H (2001). Correlated sequence-signatures as markers of protein–protein interaction1. J Mol Biol.

[16] Sun T, Zhou B, Lai L, Pei J (2017). Sequence-based prediction of protein protein interaction using a deep-learning algorithm. BMC Bioinform.

[17] You Z-H, Chan KCC, Hu P (2015). Predicting protein–protein interactions from primary protein sequences using a novel multi-scale local feature representation scheme and the random forest. PLoS ONE.

[18] Khorsand B, Savadi A, Zahiri J, Naghibzadeh M (2020). Alpha influenza virus infiltration prediction using virus-human protein–protein interaction network. Math Biosci Eng.

[19] Huang J, Ling CX (2005). Using AUC and accuracy in evaluating learning algorithms. IEEE Trans Knowl Data Eng.

[20] Lata S, Raghava GPS (2008). CytoPred: a server for prediction and classification of cytokines. Protein Eng Des Sel.

[21] Wei Q, Dunbrack RL (2013). The role of balanced training and testing data sets for binary classifiers in bioinformatics. PLoS ONE.

[22] Wei L, Bowen Z, Zhiyong C, Gao X, Liao M (2016). Exploring local discriminative information from evolutionary profiles for cytokine–receptor interaction prediction. Neurocomputing.

[23] Zou Q, Wang Z, Guan X, Liu B, Wu Y, Lin Z (2013). An approach for identifying cytokines based on a novel ensemble classifier. Biomed Res Int.

[24] Wei L, Quan Z, Minghong L, Huijuan L, Yuming Z (2016). A novel machine learning method for cytokine–receptor interaction prediction. Comb Chem High Throughput Screen.

[25] Ben-Hur A, Noble WS (2006). Choosing negative examples for the prediction of protein–protein interactions. BMC Bioinform.

[26] Jansen R, Yu H, Greenbaum D, Kluger Y, Krogan NJ, Chung S, Emili A, Snyder M, Greenblatt JF, Gerstein M (2003). A Bayesian networks approach for predicting protein–protein interactions from genomic data. Science.

[27] Jansen R, Gerstein M (2004). Analyzing protein function on a genomic scale: the importance of gold-standard positives and negatives for network prediction. Curr Opin Microbiol.

[28] Ben-Hur A, Noble WS (2005). Kernel methods for predicting protein–protein interactions. Bioinformatics.

[29] Zhang LV, Wong SL, King OD, Roth FP (2004). Predicting co-complexed protein pairs using genomic and proteomic data integration. BMC Bioinform.

[30] Tuncbag N, Gursoy A, Nussinov R, Keskin O (2011). Predicting protein–protein interactions on a proteome scale by matching evolutionary and structural similarities at interfaces using PRISM. Nat Protoc.

[31] Zahiri J, Mohammad-Noori M, Ebrahimpour R, Saadat S, Bozorgmehr JH, Goldberg T, Masoudi-Nejad A (2014). LocFuse: Human protein–protein interaction prediction via classifier fusion using protein localization information. Genomics.

[32] Launay G, Ceres N, Martin J (2017). Non-interacting proteins may resemble interacting proteins: prevalence and implications. Sci Rep..

[33] Chandola V, Banerjee A, Kumar V (2009). Anomaly detection: a survey. ACM Comput Surv.

[34] Park Y, Marcotte EM (2011). Revisiting the negative example sampling problem for predicting protein–protein interactions. Bioinformatics.

[35] Hall M, Frank E, Holmes G, Pfahringer B, Reutemann P, Witten IH (2009). The WEKA data mining software: an update. SIGKDD Explor Newsl.

[36] Witten IH, Frank E, Hall MA, Pal CJ. Chapter 10—Deep learning. In: *Data mining (fourth edition).* London: Morgan Kaufmann; 2017. p. 417–66.

[37] Nath A, Karthikeyan S (2018). Enhanced prediction of recombination hotspots using input features extracted by class specific autoencoders. J Theor Biol.

[38] Sakurada M, Yairi T. Anomaly detection using autoencoders with nonlinear dimensionality reduction. In: *Proceedings of the MLSDA 2014 2nd workshop on machine learning for sensory data analysis; Gold Coast, Australia QLD, Australia*. 2689747. London: ACM; 2014. p. 4–11.

[39] Bradley AP (1997). The use of the area under the ROC curve in the evaluation of machine learning algorithms. Pattern Recogn.

[40] Ling CX, Huang J, Zhang H. AUC: A better measure than accuracy in comparing learning algorithms. In: Xiang Y, Chaib-Draa B, editors.* Advances in artificial intelligence: 16th conference of the Canadian society for computational studies of intelligence, AI 2003, Halifax, Canada, June 11–13, 2003, Proceedings.* Berlin, Heidelberg: Springer; 2003. p. 329–41.

[41] Kubat M, Holte R, Matwin S. Learning when negative examples abound. In: van Someren M, Widmer G, editors*. Machine learning: ECML-97: 9th European conference on machine learning Prague, Czech Republic, April 23–25, 1997 Proceedings*. Berlin: Springer; 1997. p. 146–53.

[42] Nath A, Subbiah K (2016). Unsupervised learning assisted robust prediction of bioluminescent proteins. Comput Biol Med.

[43] Jo T, Japkowicz N (2004). Class imbalances versus small disjuncts. SIGKDD Explor Newsl.

[44] Han J, Kamber M, Pei J, Han J, Kamber M, Pei J (2012). 3—Data preprocessing. Data mining (Third Edition).

[45] Daszykowski M, Walczak B, Massart DL (2002). Representative subset selection. Anal Chim Acta.

