# Supplementary material for: Improved cytokine–receptor interaction prediction by exploiting the negative sample space
Source: BMC Bioinformatics. 2020 Oct 31;21:493. doi: 10.1186/s12859-020-03835-5 (PMC7603689; doi:10.1186/s12859-020-03835-5)
Supplement: Supplementary file 1 — Additional file 1. Supplementary material (docx file). [file 12859_2020_3835_MOESM1_ESM.docx]

Improved cytokine-receptor interaction prediction by exploiting the negative sample space

**Abhigyan Nath^1^, André Leier^2^**

**^1^ Department of Biochemistry, Pt. Jawahar Lal Nehru Memorial Medical College, Raipur-492001, India**

[**^1^abhigyannath01@gmail.com**](mailto:1abhigyannath01@gmail.com)

**^2^Department of Genetics and Department of Cell Developmental and Integrative Biology, School of Medicine, University of Alabama at Birmingham, AL, USA**

### ^2^ aleier@uabmc.edu

**Supplementary Material**

Description of Machine Learning Methods.

Description of Features.

Uniform Sampling Approaches.

Tables S1 – S17.

Figures S1 – S3.

List of UniProtKB Accession Numbers of Human Cytokines and Receptors.

### Description of Machine Learning Algorithms

Bagging [1], also known as bootstrap aggregation, uses sampling with replacement for drawing random subsamples that are then used for training a number of base learners. The concept of bagging is further implemented in Random Forest (RF) [2]. In RF, a group of decision trees are trained on a bagged sample from the training data. An unpruned decision tree is grown with randomly selected subsets of features at each node of the tree for optimal splitting. The outcomes of all individual decision trees are fused to form the final decision.

Nearest neighbor classifier (IBK): This group of ML algorithms uses a distance metric to determine a predefined number of nearest neighbors and then assigns the class to the test instance based on the majority vote of the nearest neighbors. Such algorithms are also known as lazy learners as no explicit model is constructed, i.e. the algorithm does not learn any type of relationship between the different features and the different classes [3, 4].

Support vector machines (SVMs) are based on structural risk minimization of statistical learning theory [5]. SVMs involve the construction of a hyperplane in a higher dimensional space for separating the different classes. For faster optimization, sequential minimization optimization (SMO) [6] is used along with radial basis function (RBF) kernel, poly kernel (PolyK) and Pearson universal kernel (PuK).

Naive Bayes (NB) and Average 1 dependence estimator (A1DE): NB is one of the simplest ML algorithms. It is assumes complete independence among the different attributes/features [7, 8]. A1DE is based on a modification of this independence assumption of NB: instead of complete independence, it takes one dependence between the attributes/features into account [9].

We have implemented all ML algorithms on the Weka ML platform [10] using default parameters.

### Description of Features

### Classical sequence-based features

**Amino acid composition (AAC)**: The simplest feature of any protein sequence is its amino acid composition, a 20-dimensional feature vector describing the frequencies of all twenty amino acid types in the given protein sequence. It is calculated by the formula

$${AAC}_{i}={N_{i}}/L ,i=1,\ldots,20,$$

where *L* is the length of the protein sequence and *N_i_* is the number of occurrences of a particular amino acid *i*.

**Dipeptide counts (DPC)**: To take the local residue order and coupling effects into account, we extracted the counts of 400 possible dipeptides yielding a feature vector of length 400.

**Property group composition (PGC)**: We used the reduced amino acid composition as described in [11] as another set of features. The reduced grouping of amino acid residues has been found useful in many protein classification systems [12-15]. The grouping is as follows (i) ***Tiny***: Ala, Cys, Gly, Ser, Thr; (ii) ***Small***: Ala, Cys, Asp, Gly, Asn, Pro, Ser, Thr, Val; (iii) ***Aliphatic***: Ile, Leu, Val; (iv) ***Non-polar***: Ala, Cys, Phe, Gly, Ile, Leu, Met, Pro,Val, Trp, Tyr; (v) ***Aromatic***: Phe, His, Trp, Tyr; (vi) ***Polar***: Asp, Glu, His, Lys, Asn, Gln, Arg, Ser, Thr; (vii) ***Charged***: Asp, Glu, His, Arg, Lys; (viii) ***Basic***: His, Lys, Arg; (ix) ***Acidic***: Asp, Glu; (x) ***Hydrophobic***: Ala, Cys, Phe, Ile, Leu, Met, Val, Trp, Tyr; (xi) ***Hydrophilic***: Asp, Glu, Lys, Asn, Gln. The 11-dimensional feature vector is calculated as follows:

$${PGC}_{i}={N_{i}}/L , i=1,\ldots,11,$$

where *L* is the length of the protein sequence and *N_i_* is the number of occurrences of a particular amino acid property group *i* in that sequence.

**Physicochemical-ngrams (PnG)**: To take the similarities of local physicochemical regions into account, we used physicochemical-n-grams (*n*= window length) as an additional feature. Specifically, using a sliding window of length 2, we calculated the physicochemical-2grams for the 11 different amino acid property groups (see PGC), giving rise to an 11-dimensional feature vector. The formula for calculating the physicochemical-2gram for property group is given as follows:

${P2G}_{p}=\sum_{i=1}^{L-1} C\left( i,i+1 \right) , p=1,\ldots,11$,

where *L* is the length of the protein sequence, *i* is the position of the first of two adjacent amino acids, and *C(i, i+1)* is a binary valued quantity. The latter is calculated as follows: If ${(a}_{i}\in S_{p}\boldsymbol{AND}a_{i+1}\in S_{p})$is true, then the value of *C(i,i+1*) is one, else zero. Here, $S_{p}$ is the set of amino acids in property group $p$ and *a_i_* and *a_i+1_* are the amino acid residues at positions *i* and *i+1* of the protein sequence, respectively*.*

**Atomic Composition (ATC)**: We have incorporated the atomic composition of Carbon (C), Hydrogen (H), Nitrogen (N), Oxygen (O), and Sulfur (S) as yet another feature set, encoded in a 5-dimensional vector.

**Physicochemical properties (PCP)**: For each protein sequence, we also calculated the mean values of six physicochemical properties that were obtained from the AAindex database [16], namely Average Volume of Residues (PONJ960101), Average Flexibility (BHAR880101), Bulkiness (ZIMJ680102), Charge (KLEP840101), Hydrophobicity (CASG920101), and Polarity (GRAR740102). We have selected only 6 physicochemical indices as they have been previously used successfully in classification and prediction tasks [12, 13, 17, 18]. Furthermore, we calculated the estimated isoelectric point and included it as an additional feature. Thus, the total length of the PCP feature vector is 7.

### Features based on evolutionary information

Features from evolutionary information are incorporated from position specific scoring matrices (PSSMs), consisting of *L* x 20 numerical values, where *L* is the length of the protein sequence. The columns of a PSSM are indexed by the 20 amino acids and the rows are indexed by the individual amino acid residues present in the protein sequence. Here, we used two types of information representations from PSSMs.

**AAC_PSSM**: This feature describes the conservation scores of individual amino acid residues in the protein sequence *P* when being mutated to any of the other amino acid residues [19]. Each entry in the PSSM is normalized using the sigmoidal function $f\left( x \right)=1/\left( 1+e^{-x} \right)$, where *x* is the original PSSM entry. Let *M* = (*M*_1_, *M*_2,_ …, *M*_20_) be the PSSM for the query protein sequence *P* of length *L*, where *M*_j_ = (*M*_1,j_, *M*_2,j_,……,*M_Lj_*)*^T^*, *j* = 1, 2 …., 20 and *T* is the transpose operator. Then, the feature vector *S*= (*S*_1_, *S*_2_, …, *S*_20_)*^T^* representing *P* is calculated as follows:

$S_{j}={\sum_{i=1}^{L} M_{i,j}}/L$.

**D-FPSSM**: This feature vector is generated by summing the entries in each of the 20 columns of the PSSM, after removing all negative scores along with those positive scores that are greater than expected by random [20]. We used the POSSUM webserver [21] to calculate AAC_PSSM and D-FPSSM. The database selected by POSSUM is Uniref50 with 3 iterations and an E-value threshold of 0.001 for BLAST [22]. A pair’s feature vector is formed by fusing the two feature vectors of the individual proteins, *FV* = (*fv*_1_, *fv*_2_).

### Uniform Sampling Approaches

Uniform sampling (Kennard Stone algorithm) is implemented and applied in two ways:

1. The KS algorithm is used to sample 203 (equal to the number of positive samples) out of 12,343 non-interacting, negative pairs. Together with the 203 positive samples, these comprise the dataset that is then randomly split into 70% training and 30% testing data.
2. The KS algorithm is used to sample 203 (equal to the number of positive samples) out of 12,343 non-interacting, negative pairs. Subsequently, the KS algorithm is also applied to the 203 negative samples to obtain 70% training (negative) data and 30% testing (negative) data. Similarly, the KS algorithm is also applied to the 203 positive samples to obtain 70% training (positive) data and 30% testing (positive) data.

Table S1: Performance evaluation metrics for the different machine learning algorithms using only amino acid composition features.

| AAC | | | | | | |
| --- | --- | --- | --- | --- | --- | --- |
|  | Sensitivity | Specificity | Accuracy | MCC | AUC | g-means |
| NB | 80.3 | 67.5 | 73.9 | 0.482 | 0.791 | 73.6 |
| A1DE | 76.8 | 60.1 | 68.5 | 0.375 | 0.668 | 67.9 |
| SMO-RBF | **84.2** | 77.8 | **81.0** | **0.622** | 0.810 | **81.0** |
| SMO-PolyK | 83.3 | 64.0 | 73.6 | 0.482 | 0.736 | 73.0 |
| SMO-PuK | 79.3 | **81.8** | 80.5 | 0.621 | 0.805 | 80.5 |
| IBK | 78.3 | 65.5 | 71.9 | 0.442 | 0.442 | 71.6 |
| Bagging | 73.9 | 74.9 | 74.4 | 0.488 | 0.808 | 74.3 |
| RF | 78.3 | 77.8 | 78.1 | 0.562 | **0.857** | 78.0 |

Table S2: Performance evaluation metrics for the different machine learning algorithms using only dipeptide composition features.

| DPC | | | | | | |
| --- | --- | --- | --- | --- | --- | --- |
|  | Sensitivity | Specificity | Accuracy | MCC | AUC | g-means |
| NB | **87.2** | 62.1 | 74.6 | 0.509 | 0.805 | 73.8 |
| A1DE | 85.2 | 71.4 | 78.3 | 0.572 | 0.859 | 77.9 |
| SMO-RBF | 27.1 | **99.0** | 63.1 | 0.376 | 0.631 | 51.7 |
| SMO-PolyK | 73.4 | 75.9 | 74.6 | 0.493 | 0.746 | 74.6 |
| SMO-PuK | 78.8 | 76.8 | 77.8 | 0.557 | 0.778 | 77.2 |
| IBK | 74.9 | 71.9 | 73.4 | 0.468 | 0.735 | 73.3 |
| Bagging | 76.8 | 73.4 | 75.1 | 0.503 | 0.843 | 75.0 |
| RF | 84.7 | 73.4 | **79.1** | **0.585** | **0.870** | **78.8** |

Table S3: Performance evaluation metrics for the different machine learning algorithms using only amino acid property group composition features.

| PGC | | | | | | |
| --- | --- | --- | --- | --- | --- | --- |
|  | Sensitivity | Specificity | Accuracy | MCC | AUC | g-means |
| NB | 58.1 | 70.4 | 64.8 | 0.297 | 0.731 | 63.9 |
| A1DE | 68.5 | 75.4 | 71.9 | 0.439 | 0.729 | 71.8 |
| SMO-RBF | 82.8 | 68.5 | 75.6 | 0.518 | 0.756 | 75.3 |
| SMO-PolyK | 76.8 | 63.5 | 70.2 | 0.408 | 0.702 | 69.8 |
| SMO-PuK | **85.2** | 75.4 | **80.3** | **0.609** | 0.803 | **80.1** |
| IBK | 76.8 | 66.0 | 71.4 | 0.431 | 0.714 | 71.1 |
| Bagging | 74.9 | 73.9 | 74.4 | 0.488 | 0.837 | 74.3 |
| RF | 78.8 | **77.3** | 78.1 | 0.562 | **0.869** | 78.0 |

Table S4: Performance evaluation metrics for the different machine learning algorithms using only physicochemical properties.

| PCP | | | | | | |
| --- | --- | --- | --- | --- | --- | --- |
|  | Sensitivity | Specificity | Accuracy | MCC | AUC | g-means |
| NB | 73.4 | 65.0 | 69.2 | 0.386 | 0.762 | 69.0 |
| A1DE | **81.8** | 69.5 | 75.6 | 0.516 | 0.721 | 75.3 |
| SMO-RBF | 77.3 | 66.0 | 71.7 | 0.436 | 0.717 | 71.4 |
| SMO-PolyK | 77.8 | 64.5 | 71.2 | 0.427 | 0.712 | 70.8 |
| SMO-PuK | 78.8 | **75.4** | 77.1 | **0.542** | 0.771 | **77.0** |
| IBK | 78.8 | 69.3 | 74.1 | 0.485 | 0.741 | 73.8 |
| Bagging | 78.3 | 64.5 | 71.4 | 0.433 | 0.808 | 71.0 |
| RF | 79.8 | 73.4 | **76.6** | 0.533 | **0.843** | 76.5 |

| P2G | | | | | | |
| --- | --- | --- | --- | --- | --- | --- |
|  | Sensitivity | Specificity | Accuracy | MCC | AUC | g-means |
| NB | 87.2 | 68.0 | 77.6 | 0.562 | 0.817 | 77.0 |
| A1DE | 90.1 | 58.6 | 74.4 | 0.514 | 0.828 | 72.6 |
| SMO-RBF | 84.2 | 72.4 | 78.3 | 0.571 | 0.783 | 78.0 |
| SMO-PolyK | 80.3 | 72.9 | 76.8 | 0.533 | 0.766 | 76.5 |
| SMO-PuK | **91.1** | 75.9 | **83.5** | **0.678** | 0/835 | **83.1** |
| IBK | 83.7 | 73.9 | 78.8 | 0.579 | 0.788 | 78.6 |
| Bagging | 88.7 | 73.9 | 81.3 | 0.633 | 0.874 | 80.9 |
| RF | 87.7 | **76.8** | 82.3 | 0.649 | **0.904** | 82.0 |

Table S5: Performance evaluation metrics for the different machine learning algorithms using only physicochemical-2grams composition features.

| ATC | | | | | | |
| --- | --- | --- | --- | --- | --- | --- |
|  | Sensitivity | Specificity | Accuracy | MCC | AUC | g-means |
| NB | 92.1 | 56.7 | 74.4 | 0.522 | 0.787 | 72.2 |
| A1DE | 89.2 | 51.2 | 70.2 | 0.437 | 0.742 | 67.6 |
| SMO-RBF | **94.6** | 65.5 | 80.0 | 0.628 | 0.800 | 78.7 |
| SMO-PolyK | 93.6 | 63.5 | 78.6 | 0.599 | 0.786 | 77.0 |
| SMO-PuK | 93.1 | 65.5 | 80.3 | 0.634 | 0.803 | 78.0 |
| IBK | 88.7 | 78.3 | 83.5 | 0.674 | 0.835 | 83.3 |
| Bagging | 90.1 | 71.9 | 81.0 | 0.631 | 0.872 | 80.5 |
| RF | 89.2 | **78.8** | **84.0** | **0.683** | **0.898** | **84.0** |

Table S6: Performance evaluation metrics for the different machine learning algorithms using only atomic composition features.

Table S7: Performance evaluation metrics for the different machine learning algorithms using the combined features of atomic composition and physicochemical-2grams.

| ATC+P2G | | | | | | |
| --- | --- | --- | --- | --- | --- | --- |
|  | Sensitivity | Specificity | Accuracy | MCC | AUC | g-means |
| NB | 89.7 | 64.5 | 77.1 | 0.560 | 0.814 | 76.0 |
| A1DE | **94.6** | 55.2 | 74.9 | 0.541 | 0.825 | 72.3 |
| SMO-RBF | 91.1 | 71.4 | 81.3 | 0.638 | 0.813 | 80.6 |
| SMO-PolyK | 84.2 | 72.9 | 78.6 | 0.575 | 0.786 | 78.3 |
| SMO-PuK | 91.1 | **76.4** | **83.7** | **0.682** | 0.837 | **83.4** |
| IBK | 85.7 | 74.9 | 80.3 | 0.610 | 0.803 | 80.1 |
| Bagging | 90.1 | 75.9 | 83.0 | 0.667 | 0.884 | 82.6 |
| RF | 88.7 | 74.9 | 81.8 | 0.642 | **0.912** | 81.5 |

| AAC_PSSM | | | | | | |
| --- | --- | --- | --- | --- | --- | --- |
|  | Sensitivity | Specificity | Accuracy | MCC | AUC | g-means |
| NB | 81.8 | 68.0 | 74.9 | 0.502 | 0.813 | 74.6 |
| A1DE | 86.7 | 76.4 | 81.5 | 0.634 | 0.906 | 81.3 |
| SMO-RBF | 87.2 | 86.7 | 86.9 | 0.739 | 0.869 | 86.9 |
| SMO-PolyK | 87.2 | 77.3 | 82.3 | 0.648 | 0.823 | 82.1 |
| SMO-PuK | 86.2 | **88.2** | **87.2** | **0.744** | 0.872 | **87.2** |
| IBK | **88.7** | 80.3 | 84.5 | 0.692 | 0.845 | 84.3 |
| Bagging | **88.7** | 80.8 | 84.7 | 0.697 | 0.913 | 84.6 |
| RF | **88.7** | 83.7 | 86.2 | 0.725 | **0.935** | 86.1 |

Table S8: Performance evaluation metrics for the different machine learning algorithms using only AAC_PSSM features.

Table S9: Performance evaluation metrics for the different machine learning algorithms using only D-FPSSM features.

| D-FPSSM | | | | | | |
| --- | --- | --- | --- | --- | --- | --- |
|  | Sensitivity | Specificity | Accuracy | MCC | AUC | g-means |
| NB | **97.5** | 48.8 | 73.2 | 0.530 | 0.832 | 68.9 |
| A1DE | 93.6 | 71.9 | 82.8 | 0.671 | 0.869 | 81.8 |
| SMO-RBF | 92.6 | 81.3 | **86.9** | **0.744** | 0.869 | **86.7** |
| SMO-PolyK | 90.6 | 75.4 | 83.0 | 0.668 | 0.830 | 82.6 |
| SMO-PuK | 89.2 | **83.7** | 86.5 | 0.730 | 0.865 | 86.4 |
| IBK | 84.2 | 75.4 | 79.8 | 0.598 | 0.798 | 79.6 |
| Bagging | 89.2 | **83.7** | 86.5 | 0.730 | 0.865 | 86.4 |
| RF | 89.7 | 82.3 | 86.0 | 0.721 | **0.939** | 86.0 |

Table S10: Performance evaluation metrics for the different machine learning algorithms using AAC_PSSM and D-FPSSM features.

| AAC_PSSM+D-FPSSM | | | | | | |
| --- | --- | --- | --- | --- | --- | --- |
|  | Sensitivity | Specificity | Accuracy | MCC | AUC | g-means |
| NB | **95.1** | 61.6 | 78.3 | 0.601 | 0.835 | 76.5 |
| A1DE | 94.1 | 77.3 | 85.7 | 0.725 | 0.912 | 85.2 |
| SMO-RBF | 92.6 | 85.7 | **89.2** | **0.785** | 0.892 | **89.0** |
| SMO-PolyK | 94.6 | 75.9 | 85.2 | 0.717 | 0.852 | 84.7 |
| SMO-PuK | 89.2 | **88.8** | 85.2 | 0.773 | 0.887 | 88.9 |
| IBK | 89.2 | 81.3 | 85.2 | 0.707 | 0.852 | 85.1 |
| Bagging | 86.2 | 80.8 | 83.5 | 0.671 | 0.916 | 83.4 |
| RF | 90.1 | 83.3 | 86.7 | 0.736 | **0.935** | 86.6 |

Table S11: Performance evaluation metrics for the different machine learning algorithms using all features.

| All features | | | | | | |
| --- | --- | --- | --- | --- | --- | --- |
|  | Sensitivity | Specificity | Accuracy | MCC | AUC | g-means |
| NB | 89.2 | 71.9 | 80.5 | 0.620 | 0.832 | 79.6 |
| A1DE | **94.6** | 72.9 | **83.7** | **0.691** | 0.890 | **83.0** |
| SMO-RBF | 22.7 | **99.5** | 61.1 | 0.346 | 0.611 | 47.5 |
| SMO-PolyK | 72.4 | 74.4 | 73.4 | 0.468 | 0.734 | 73.3 |
| SMO-PuK | 78.8 | 78.8 | 78.8 | 0.567 | 0.788 | 78.8 |
| IBK | 80.3 | 67.0 | 73.6 | 0.477 | 0.737 | 73.3 |
| Bagging | 87.2 | 76.4 | 81.8 | 0.639 | 0.894 | 81.6 |
| RF | 85.2 | 78.3 | 81.8 | 0.637 | **0.903** | 81.6 |

Table S12: Accuracy for all model algorithm and feature set configurations.

| **Accuracy** | AAC | DPC | PGC | PCP | P2G | ATC | AAC_PSSM | D-FPSSM | AAC_PSSM+ D-FPSSM | ATC +P2G | All | ATC+P2G+AAC_ PSSM+D-FPSSM |
| --- | --- | --- | --- | --- | --- | --- | --- | --- | --- | --- | --- | --- |
| NB | 73.9 | 74.6 | 64.8 | 69.2 | 77.6 | 74.4 | 74.9 | 73.2 | 78.3 | 77.1 | 80.5 | **81.3** |
| A1DE | 68.5 | 78.3 | 71.9 | 75.6 | 74.4 | 70.2 | 81.5 | 82.8 | **85.7** | 74.9 | 83.7 | 82.3 |
| SMO-RBF | 81.0 | 63.1 | 75.6 | 71.7 | 78.3 | 80.0 | 86.9 | 86.9 | 89.2 | 81.3 | 61.1 | **90.1** |
| SMO-PolyK | 73.6 | 74.6 | 70.2 | 71.2 | 76.8 | 78.6 | 82.3 | 83.0 | **85.2** | 78.6 | 73.4 | 85.0 |
| SMO-PuK | 80.5 | 77.8 | 80.3 | 77.1 | 83.5 | 80.3 | 87.2 | 86.5 | 85.2 | 83.7 | 78.8 | **89.4** |
| IBK | 71.9 | 73.4 | 71.4 | 74.1 | 78.8 | 83.5 | 84.5 | 79.8 | 85.2 | 80.3 | 73.6 | **87.9** |
| Bag-ging | 74.4 | 75.1 | 74.4 | 71.4 | 81.3 | 81.0 | 84.7 | **86.5** | 83.5 | 83.0 | 81.8 | 82.8 |
| RF | 78.1 | 79.1 | 78.1 | 76.6 | 82.3 | 84.0 | 86.2 | 86.0 | **86.7** | 81.8 | 81.8 | 85.2 |

Table S13: Autoencoder model parameters that were explored for selecting the best model.

| No. | Model Parameters | MSE | RMSE |
| --- | --- | --- | --- |
| 1 | *Hidden layers=1000,500,100,50,20,10*  *activation function=Tanh,*  *l2=0.0001,*  *epochs=1000* | 0.0051 | 0.071 |
| 2 | *Hidden layers=1000,500,100,50,20,10*  *activation function=Rectifier*  *l2=0.0001,*  *epochs=1000* | 0.0460 | 0.214 |
| 3 | *Hidden layers=10,20,100,500,1000*  *activation function=Tanh*  *l2=0.0001,*  *epochs=1000* | 0.0471 | 0.217 |
| 4 | *Hidden layers=100,500,1000*  *activation function=Tanh*  *l2=0.0001,*  *epochs=1000* | 0.0017 | 0.042 |
| 5 | *Hidden layers=1000,500,100*  *activation function=Tanh*  *l2=0.0001,*  *epochs=1000* | 0.00067 | 0.0258 |
| 6 | *Hidden layers=500,100,500*  *activation function=Tanh*  *l2=0.0001,*  *epochs=1000* | 0.0044 | 0.0664 |
| 7 | *Hidden layers=1000,500,100,50*  *activation function=Tanh*  *l2=0.0001,*  *epochs=1000* | 0.0010 | 0.0322 |
| 8 | *Hidden layers=100,500,100*  *activation function=Tanh*  *l2=0.0001,*  *epochs=1000* | 0.00068 | 0.0261 |
| 9 | *Hidden layers=1000,500,100,50,20*  *activation function=Tanh*  *l2=0.0001,*  *epochs=1000* | 0.0027 | 0.0523 |

Table S14: Distribution of interacting and non-interacting protein pairs into different bins (based on the reconstruction error values) and their counts.

|  | **Interacting proteins** | | **Non-interacting proteins** | |
| --- | --- | --- | --- | --- |
| **No.** | **Error interval** | **counts** | **Error interval** | **counts** |
| 1 | [0.000, 0.001] | 183 | [0.000,0.001] | 2542 |
| 2 | (0.001,0.002] | 16 | (0.001,0.002] | 6166 |
| 3 | (0.002,0.003] | 2 | (0.002,0.003] | 2340 |
| 4 | (0.003,0.004] | 0 | (0.003,0.004] | 662 |
| 5 | (0.004,0.005] | 1 | (0.004,0.005] | 205 |
| 6 | (0.005,0.006] | 0 | (0.005,0.006] | 104 |
| 7 | (0.006,0.007] | 1 | (0.006,0.007] | 79 |
| 8 |  |  | (0.007,0.008] | 51 |
| 9 |  |  | (0.008,0.010] | 48 |
| 10 |  |  | (0.010,0.012] | 22 |
| 11 |  |  | (0.012,0.014] | 36 |
| 12 |  |  | (0.014,0.016] | 29 |
| 13 |  |  | (0.016,0.018] | 14 |
| 14 |  |  | (0.018,0.020] | 9 |
| 15 |  |  | (0.020,0.022] | 3 |
| 16 |  |  | (0.022,0.024] | 8 |
| 17 |  |  | (0.024,0.026] | 4 |
| 18 |  |  | (0.026,0.028] | 14 |
| 19 |  |  | (0.028,0.030] | 6 |
| 20 |  |  | (0.030,0.032] | 0 |
| 21 |  |  | (0.032,0.034] | 0 |
| 22 |  |  | (0.034,0.036] | 0 |
| 23 |  |  | (0.036,0.038] | 0 |
| 24 |  |  | (0.038,0.040] | 1 |

Table S15: Performance evaluation metrics for SMO-RBF on different samples of non-interacting protein pairs grouped according to their reconstruction error after being projected through an autoencoder that had been trained on interacting protein pairs.

|  | **Reconstruction error ≤0.001** | | | | | |
| --- | --- | --- | --- | --- | --- | --- |
|  | Sensitivity | Specificity | Accuracy | MCC | AUC | g-means |
| 1 | 64.0 | 66.5 | 65.3 | 0.306 | 0.653 | 65.2 |
| 2 | 59.6 | 68.0 | 63.8 | 0.277 | 0.638 | 63.6 |
| 3 | 59.6 | 59.6 | 59.6 | 0.192 | 0.596 | 59.6 |
| 4 | **67.0** | 65.0 | **66.0** | 0.320 | **0.660** | **66.0** |
| 5 | 63.5 | **68.5** | **66.0** | **0.321** | **0.660** | **66.0** |
| average | 62.7 | 65.5 | 64.1 | 0.283 | 0.641 | 64.1 |
|  | **Reconstruction error ≤0.003** | | | | | |
|  | Sensitivity | Specificity | Accuracy | MCC | AUC | g-means |
| 1 | 82.3 | 70.9 | 76.6 | 0.535 | 0.766 | 76.3 |
| 2 | 74.4 | 70.0 | 72.2 | 0.444 | 0.722 | 72.2 |
| 3 | 76.8 | **76.8** | 76.8 | 0.537 | 0.768 | 76.8 |
| 4 | 78.8 | 73.4 | 76.1 | 0.523 | 0.761 | 76.0 |
| 5 | **86.2** | 69.0 | **77.6** | **0.560** | **0.776** | **77.1** |
| average | 79.7 | 72.0 | 75.8 | 0.519 | 0.758 | 75.6 |
|  | **Reconstruction error>0.003** | | | | | |
|  | Sensitivity | Specificity | Accuracy | MCC | AUC | g-means |
| 1 | 94.1 | **99.5** | **96.8** | **0.937** | **0.968** | **96.7** |
| 2 | 93.6 | 99.0 | 96.3 | 0.927 | 0.963 | 96.2 |
| 3 | 93.6 | 97.5 | 95.6 | 0.912 | 0.956 | 95.5 |
| 4 | **94.6** | 98.5 | 96.6 | 0.932 | 0.966 | 96.5 |
| 5 | 94.1 | 97.0 | 95.6 | 0.912 | 0.956 | 95.5 |
| average | 94.0 | 98.3 | 96.1 | 0.924 | 0.961 | 96.1 |

Table S16: Performance evaluation metrics for the different machine learning algorithms with different training/testing sets generated by applying the uniform selection strategy #1 to the original set of 12,343 negatives, producing a subset of 203 negatives that were then (like the positive dataset) randomly split into training and testing sets (70:30 ratio). See additional explanation on Uniform Sampling Approaches in the Suppl. Material.

|  | | | | | | |
| --- | --- | --- | --- | --- | --- | --- |
|  | Sensitivity | Specificity | Accuracy | MCC | AUC | g-means |
| NB | 82.8 | 68.8 | 75.4 | 0.518 | 0.820 | 75.4 |
| A1DE | **86.2** | 56.3 | 70.5 | 0.442 | 0.816 | 69.6 |
| SMO-RBF | 82.8 | 78.1 | **80.3** | **0.608** | 0.804 | **80.4** |
| SMO-PolyK | 81.0 | 65.6 | 73.0 | 0.470 | 0.733 | 72.8 |
| SMO-PuK | 79.3 | 81.3 | **80.3** | 0.606 | 0.803 | 80.2 |
| IBK | 82.8 | 65.6 | 73.8 | 0.489 | 0.742 | 73.6 |
| Bagging | 70.7 | **85.9** | 78.7 | 0.575 | **0.890** | 77.9 |
| RF | 79.3 | 81.3 | **80.3** | 0.606 | 0.874 | 80.2 |

Table S17: Performance evaluation metrics for the different machine learning algorithms with different testing sets generated by applying the uniform selection strategy #2. See additional explanation on Uniform Sampling Approaches in the Suppl. Material).

|  | | | | | | |
| --- | --- | --- | --- | --- | --- | --- |
|  | Sensitivity | Specificity | Accuracy | MCC | AUC | g-means |
| NB | **100** | 47.5 | 73.8 | 0.558 | 0.865 | 68.9 |
| A1DE | 98.4 | 59.0 | 78.7 | 0.624 | 0.919 | 76.1 |
| SMO-RBF | **100** | **79.8** | **86.9** | **0.764** | 0.869 | **89.3** |
| SMO-PolyK | **100** | 55.7 | 77.9 | 0.622 | 0.779 | 74.6 |
| SMO-PuK | **100** | 72.1 | 86.1 | 0.751 | 0.861 | 84.9 |
| IBK | **100** | 54.1 | 77.0 | 0.609 | 0.770 | 73.7 |
| Bagging | 96.7 | 65.6 | 81.1 | 0.651 | 0.894 | 79.6 |
| RF | 96.7 | 60.7 | 78.7 | 0.615 | **0.956** | 76.6 |

Fig. S1 Schematic representation of the current approach

**Dataset**

**Feature Extraction**

**Random Sampling**

K-means Sampling

Uniform Sampling

Machine Learning Algorithms

**Autoencoder based negative dataset analysis**

**Fusion of Best Feature Set**

Machine Learning Algorithms

Figure S2 Reconstruction error (sorted in increasing order) (A, C) and histogram (B, D) of the reconstruction error of interacting (A, B) and non-interacting (C, D) proteins.


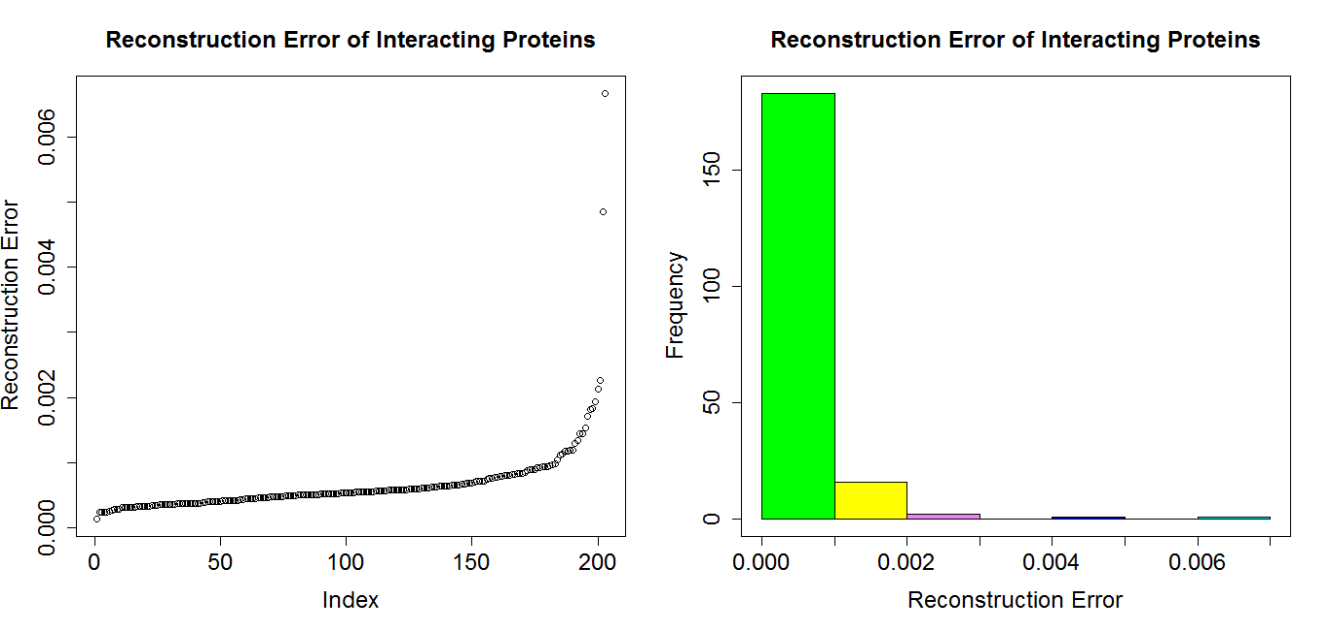


**A B**


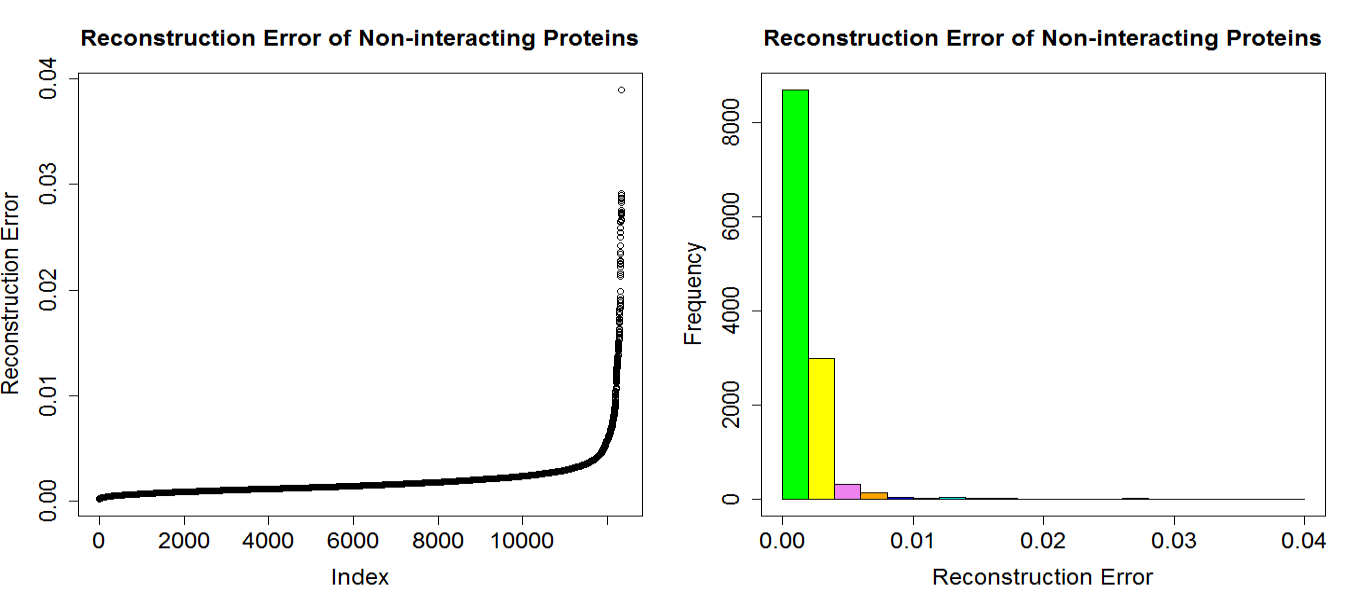


**C D**

Figure S3: Distribution of non-interacting proteins in different clusters for K=203.

List of UniProtKB Accession Numbers (2017_11) of human cytokines and receptors

| Q04771.1 | P41597.1 | Q92838.2 | Q9UHF5.1 | P10145.1 | Q9UBN6.1 |
| --- | --- | --- | --- | --- | --- |
| P36896.1 | P51677.1 | Q9UNE0.1 | Q9H293.1 | P25024.2 | Q9Y6Q6.1 |
| P27037.1 | P51679.1 | P01133.2 | Q96F46.2 | P25025.2 | O00300.3 |
| Q13705.3 | P51681.1 | P00533.2 | Q9NRM6.2 | P15248.1 | Q9NP84.1 |
| P03971.3 | P51684.2 | P01588.1 | Q14116.1 | Q01113.3 | O14836.1 |
| Q16671.1 | P32248.2 | P19235.1 | Q13478.1 | P08476.2 | Q96RJ3.1 |
| P32302.1 | P51685.1 | P25445.1 | Q9UHD0.2 | P09529.2 | Q92956.3 |
| P12643.1 | P51686.2 | P48023.1 | P01583.1 | P55103.1 | Q02223.2 |
| P18075.1 | P25942.1 | P17948.2 | P01584.2 | P58166.1 | Q9Y5U5.1 |
| P36894.2 | P29965.1 | P36888.2 | P14778.1 | P35968.2 | P19438.1 |
| O00238.1 | Q9UBD9.1 | P49771.1 | P27930.1 | P10721.1 | P20333.3 |
| Q13873.2 | P26441.1 | P43026.3 | P60568.1 | P21583.1 | P43489.1 |
| O00590.2 | P26992.2 | P01241.2 | Q9NYY1.2 | P41159.1 | O95407.1 |
| P22362.1 | P09603.2 | P01242.3 | Q9UHF4.2 | P48357.2 | P26842.2 |
| P51671.1 | P07333.2 | P10912.1 | Q9HBE4.2 | P42702.1 | P28908.1 |
| Q99616.1 | P04141.1 | P01562.1 | Q9HBE5.1 | P01374.2 | Q07011.1 |
| Q16627.1 | P15509.1 | P17181.3 | Q9GZX6.1 | Q06643.1 | P50591.1 |
| Q16663.2 | P32927.2 | P48551.1 | Q8N6P7.1 | P36941.1 | O14788.1 |
| O15467.1 | P09919.1 | P01574.1 | Q969J5.1 | P40238.1 | O43508.1 |
| Q92583.1 | Q99062.1 | Q86WN2.1 | Q9NPF7.1 | P13725.2 | O75888.1 |
| Q99731.1 | Q16619.1 | P01579.1 | Q5VWK5.3 | Q99650.1 | Q9Y275.1 |
| P13500.1 | P78423.1 | P15260.1 | Q13007.1 | P01127.1 | O43557.2 |
| P78556.1 | P49238.1 | P38484.2 | Q8IZJ0.1 | Q9NRA1.2 | Q9UNG2.2 |
| O00585.1 | P09341.1 | Q9P0W0.2 | Q8IZI9.2 | P16234.1 | P23510.1 |
| O00626.2 | P02778.2 | P05000.2 | Q8IU57.1 | P09619.1 | P32970.2 |
| P55773.3 | O14625.1 | P22301.1 | Q8IU54.1 | Q8TD55.1 | P32971.1 |
| O00175.2 | P48061.1 | Q13651.2 | P01589.1 | P02775.3 | P41273.1 |
| O15444.2 | O43927.1 | Q08334.2 | P14784.1 | P01236.1 | P07202.4 |
| Q9Y258.1 | Q9H2A7.4 | P20809.1 | P08700.2 | P16471.1 | Q969D9.1 |
| Q9Y4X3.1 | P19875.1 | Q14626.2 | P26951.1 | P01137.2 | P15692.2 |
| Q9NRJ3.1 | P19876.1 | P29459.2 | P05112.1 | P61812.1 | P49765.2 |
| P10147.1 | P42830.1 | P42701.1 | P24394.1 | P10600.1 | P49767.1 |
| P13236.1 | P80162.4 | Q99665.1 | P05113.1 | P36897.1 | P47992.1 |
| P13501.3 | Q07325.1 | P35225.2 | Q01344.2 | P37173.2 | Q9UBD3.1 |
| P80098.3 | P49682.2 | P78552.1 | P05231.1 | P01375.1 | P46094.1 |
| P80075.2 | P61073.1 | P40933.1 | P08887.1 | O00220.3 |  |
| P32246.1 | O00574.1 | Q13261.1 | P13232.1 | O14763.2 |  |
| P46092.3 | Q16570.3 | Q16552.1 | P16871.2 | O14798.3 |  |

References

1. Breiman L: **Bagging Predictors**. *Machine Learning* 1996, **24**(2):123-140.

2. Breiman L: **Random Forests**. *Machine Learning* 2001, **45**(1):5-32.

3. Larose DT: **Discovering Knowledge in Data: An Introduction to Data Mining**: Wiley-Interscience; 2004.

4. Witten IH, Frank E, Hall MA: **Data Mining: Practical Machine Learning Tools and Techniques**: Morgan Kaufmann Publishers Inc.; 2011.

5. Vapnik VN: **The nature of statistical learning theory**: Springer-Verlag New York, Inc.; 1995.

6. Platt JC: **Fast training of support vector machines using sequential minimal optimization**. In: *Advances in kernel methods.* Edited by Bernhard S, lkopf, Christopher JCB, Alexander JS: MIT Press; 1999: 185-208.

7. John GH, Langley P: **Estimating continuous distributions in Bayesian classifiers**. In: *Proceedings of the Eleventh conference on Uncertainty in artificial intelligence; Montr&#233;al, Qu&#233;, Canada*. 2074196: Morgan Kaufmann Publishers Inc. 1995: 338-345.

8. Rish I: **An empirical study of the naive Bayes classifier**. In: *IJCAI 2001 workshop on empirical methods in artificial intelligence: 2001*. Erklärung von Naive Bayes: 41--46.

9. Webb GI, Boughton JR, Wang Z: **Not So Naive Bayes: Aggregating One-Dependence Estimators**. *Mach Learn* 2005, **58**(1):5-24.

10. Hall M, Frank E, Holmes G, Pfahringer B, Reutemann P, Witten IH: **The WEKA data mining software: an update**. *SIGKDD Explor Newsl* 2009, **11**(1):10-18.

11. Nath A, Chaube R, Subbiah K: **An insight into the molecular basis for convergent evolution in fish antifreeze Proteins**. *Computers in Biology and Medicine* 2013, **43**(7):817-821.

12. Nath A, Karthikeyan S: **Enhanced identification of β-lactamases and its classes using sequence, physicochemical and evolutionary information with sequence feature characterization of the classes**. *Computational Biology and Chemistry* 2017, **68**(Supplement C):29-38.

13. Nath A: **Insights into the sequence parameters for halophilic adaptation**. *Amino Acids* 2016, **48**(3):751-762.

14. Nath A, Karthikeyan S: **Enhanced Prediction and Characterization of CDK Inhibitors Using Optimal Class Distribution**. *Interdisciplinary Sciences: Computational Life Sciences* 2017, **9**(2):292-303.

15. Nath A, Subbiah K: **Probing an optimal class distribution for enhancing prediction and feature characterization of plant virus-encoded RNA-silencing suppressors**. *3 Biotech* 2016, **6**(1):93.

16. Kawashima S, Kanehisa M: **AAindex: amino acid index database**. *Nucleic acids research* 2000, **28**(1):374-374.

17. Yadav A, Sahu R, Nath A: **A representation transfer learning approach for enhanced prediction of growth hormone binding proteins**. *Computational Biology and Chemistry* 2020, **87**:107274.

18. Nath A: **Prediction and molecular insights into fungal adhesins and adhesin like proteins**. *Computational Biology and Chemistry* 2019, **80**:333-340.

19. Liu T, Zheng X, Wang J: **Prediction of protein structural class for low-similarity sequences using support vector machine and PSI-BLAST profile**. *Biochimie* 2010, **92**(10):1330-1334.

20. Zahiri J, Yaghoubi O, Mohammad-Noori M, Ebrahimpour R, Masoudi-Nejad A: **PPIevo: Protein–protein interaction prediction from PSSM based evolutionary information**. *Genomics* 2013, **102**(4):237-242.

21. Wang J, Yang B, Revote J, Leier A, Marquez-Lago TT, Webb G, Song J, Chou K-C, Lithgow T: **POSSUM: a bioinformatics toolkit for generating numerical sequence feature descriptors based on PSSM profiles**. *Bioinformatics* 2017, **33**(17):2756-2758.

22. Altschul SF, Gish W, Miller W, Myers EW, Lipman DJ: **Basic local alignment search tool**. *Journal of Molecular Biology* 1990, **215**(3):403-410.
